# Sex difference in the association of obesity with personal or social background among urban residents in Japan

**DOI:** 10.1371/journal.pone.0242105

**Published:** 2020-11-25

**Authors:** Shun-ichiro Asahara, Hiroshi Miura, Wataru Ogawa, Yoshikazu Tamori

**Affiliations:** 1 Division of Creative Health Promotion, Department of Social/Community Medicine and Health Science, Kobe University Graduate School of Medicine, Kobe, Japan; 2 Division of Diabetes and Endocrinology, Department of Internal Medicine, Kobe University Graduate School of Medicine, Kobe, Japan; Bradford Institute for Health Research, UNITED KINGDOM

## Abstract

The development of obesity is influenced by genetic and environmental factors and is associated with a variety of health problems. To gain insight into environmental factors that contribute to obesity, we analyzed the relation of personal or social background to obesity in men and women separately with the use of data from a community-based questionnaire survey of 5425 residents aged 20 to 64 years of Kobe, a representative large city in Japan. Obesity and normal weight were defined as a body mass index (BMI) of ≥25 and of ≥ 18.5 and < 25 kg/m^2^, respectively, according to the diagnostic criteria of the Japan Society for the Study of Obesity. The personal or social background factors examined included marital status, family structure, employment, household income, residence type, welfare enrollment, economic conditions of current life, educational level, extracurricular activity in school, living conditions at 15 years of age, and childhood adversity. We found that the prevalence of obesity was 27.2% and 10.6% in men and women, respectively. Among women, unmarried status, a low household income, welfare enrollment, difficult current economic conditions, a low educational level, and childhood adversity were associated with obesity, whereas none of the personal or social background factors examined were associated with obesity in men. Our results suggest that the development of obesity in women is strongly influenced by personal or social background, and such factors should be taken into consideration in the management of this condition in women.

## Introduction

Obesity contributes to the development of not only metabolic and cardiovascular conditions but also health problems such as respiratory, hepatic, gynecologic, and locomotor disorders [1], thereby leading to a decline in quality and expectancy of life. Obesity can also give rise to increased health care costs, unemployment, social disadvantage, and reduced economic productivity, thus imposing a socioeconomic burden [2–4]. The prevention and treatment of obesity are therefore important for the health not only of individuals but also of society and the economy.

The prevalence of obesity and the overweight state is increasing globally. The proportion of adults with a body mass index (BMI) of ≥25 kg/m^2^ was >35% for both sexes worldwide in 2013 [5]. In Japan, ~30% of adult men and 20% of adult women have a BMI of ≥25 kg/m^2^ [6]. In spite of the increasing demand for the care of obese individuals, there are few effective medications for obesity, and psychotherapy and behavioral therapy play central roles in the management of this condition.

The development of obesity or overweight is related to socioeconomic status, having been found to be associated with factors such as education, occupation, and income [7–10]. It is thus important to understand the social background of affected individuals in order to prevent and provide care for obesity and its sequelae. However, information on the personal and social background of obese individuals is limited—with a few studies having investigated the relation of a small number of life factors to obesity in Japan [11, 12].

To obtain further insight into the role of environmental factors in the development of obesity, we have now investigated the relation of various aspects of personal and social background to obesity by analysis of a questionnaire survey of health and life conducted among the citizens of Kobe, a representative urban area in Japan.

## Materials and methods

### Subjects, questionnaire, and ethical approval

This study was based on analysis of the results of a questionnaire conducted among the residents of Kobe by the city administration to grasp the health and life conditions of citizens in 2018. Kobe is the 7th largest city with a population of ~1.5 million in Japan. The proportions of the population under the age of 15, between 15 and 64 years of age, and over the age of 65 in Kobe are 12%, 61%, and 27%, respectively (2015). Such demographic composition is similar to the mean one of Tokyo, Osaka, and Nagoya (12%, 64%, and 24%) which are representative large cities in Japan. These findings imply that Kobe is a representative large city not only in terms of the city size but the demographics in Japan. The Kobe city area is divided into 78 districts, each with a population of ~20,000, for administrative purposes concerning nursing care for senior citizens. The city office randomly selected 20,000 citizens aged 20 to 64 years in such a way as to reflect the distribution of age and population in the 78 districts, and sent them a questionnaire comprising 46 questions that addressed health and life status (https://www.city.kobe.lg.jp/life/health/kenkousouzoutoshi/houkokusyo0215.pdf). Answers to the questionnaire were returned by 6666 residents (response rate of 33.3%), and permission for the use of the questionnaire answers in this research was obtained from 5630 individuals. We excluded 205 of these latter individuals because of missing information regarding age, sex, or BMI. The 46 questions of the questionnaire were divided into six categories concerning personal and social background; current health condition; diet, exercise, rest, and dental health; tobacco and alcohol use; health checkups and cancer screening; and participation in community activities and acquaintances. The category covering personal and social background included 14 questions (Q1-Q14, S1 and S2 Appendices), of which we excluded Q5, which related to the type of health insurance, given that this does not appear to be related to obesity. For this study, high, medium, and low household incomes were defined as ≥JY6,000,000, ≥JY2,000,000, and <JY2,000,000, respectively, according to the previous study conducted in Japan [12]. A high education level was defined as university attendance and beyond. Estimation of both current economic situation and living conditions at age 15 years relative to that of the general public was based on the subjective judgment of the participants. In the present study, “middle” with regard to living conditions at 15 years of age included the selections of “upper middle,” “middle,” and “lower middle” in the original questionnaire (Q13, S1 and S2 Appendices). Childhood adversity was defined as encompassing the following events: death of a parent, divorce of parents, a parent with mental illness, a father who was violent toward the mother, injury as a result of a violent blow from a parent, inadequate food or clothing in daily life, emotional trauma caused by insults or comments of a parent, and economic difficulties. This study was conducted in accordance with the Declaration of Helsinki and its amendments, and it was approved by the Ethics Committees of Kobe University Hospital (approval number B190029) and Kobe City (approval number Gan-3). All participants provided written informed consent.

### Data analysis

BMI was calculated by dividing weight (kg) by the square of height (m), both of which were derived from self-reported data in the questionnaire. Given that Japanese are at higher risk of developing diabetes or heart disease even at a low BMI compared with Caucasians [13], the diagnostic criteria of the Japan Society for the Study of Obesity define obesity as a BMI of ≥25 kg/m^2^ [14, 15], a definition that we adopted in the current study. This definition encompasses both the conditions of overweight (BMI of ≥25 kg/m^2^) and obesity (BMI of ≥30 kg/m^2^) according to the criteria of the World Health Organization [15, 16]. Normal weight was defined as 18.5 kg/m^2^ ≤ BMI < 25 kg/m^2^.

### Statistical analysis

Data were analyzed separately according to sex. Categorical data are presented as number (percentage) and were analyzed with the chi-square test. A stepwise procedure was adopted to determine factors for adjustment in logistic regression analysis. Binomial logistic regression models were used to calculate odds ratios (ORs) and 95% confidence intervals (CIs) for the prevalence of personal or social background factors among individuals of normal weight versus those with obesity. All reported p values are two-tailed. A p value of <0.05 was considered statistically significant. All statistical analysis was performed with SPSS version 26 for Windows (SPSS, Chicago, IL, USA).

## Results

### Characteristics of study subjects

Among the 5425 study subjects (2276 men, 3149 women), 954 (620 men, 334 women) and 3867 (1558 men, 2309 women) individuals were obese or of normal weight, respectively. The basic characteristics of these individuals are shown in Table 1. The mean ± SD age of the obese and normal-weight subjects was 45.0 ± 11.8 and 42.5 ± 12.5 years, respectively. The mean ± SD BMI of obese and normal-weight subjects was 27.8 ± 2.7 and 21.4 ± 1.7 kg/m^2^, respectively.

**Table 1:** Characteristics of study subjects.

	All		Men		Women	
	Obese	Normal-weight	Obese	Normal-weight	Obese	Normal-weight
N	954	3867	620	1558	334	2309
Age (Mean)	45.0 ± 11.8	42.5 ± 12.5	44.8 ± 11.8	43.1 ± 12.9	45.3 ± 11.7	42.0 ± 12.1
Age (Median)	46 (36.0–55.0)	43 (32.0–53.0)	45 (36.0–55.0)	43 (32.0–54.0)	46 (37.0–55.0)	43 (32.0–51.0)
BMI (Mean)	27.8 ± 2.7	21.4 ± 1.7	27.7 ± 2.4	22.0 ± 1.7	28.0 ± 3.1	21.0 ± 1.7
BMI (Median)	27 (25.8–29.0)	21.3 (20.0–22.8)	27 (25.8–29.0)	22 (20.7–23.5)	27 (25.9–29.0)	20.8 (19.7–22.2)

Regarding age and BMI, data are presented as means ± SD and medians (25%–75% range).

### Sex-specific differences in personal or social background between obese and normal-weight subjects

The prevalence of obesity was 27.2% and 10.6% in men and women, respectively. Given that this difference was statistically significant (p < 0.001, chi-squared test), we examined potential differences in personal or social background between normal-weight and obese subjects for men and women separately with the chi-square test. Among men, a significant difference between normal-weight and obese subjects was apparent only for age distribution (p = 0.001) (Table 2). Among women, however, significant differences were detected between normal-weight and obese subjects for all examined factors with the exception of marital status, family structure (number of household members), and residence type (Table 2).

**Table 2:** Sex-specific difference in personal or social background between obese and normal-weight subjects.

Variables	Men			Women		
	Normal-weight	Obese	*P* value	Normal-weight	Obese	*P* value
**Age (years)**						
20 ~	294 (18.9)	80 (12.9)	0.001	462 (20.0)	42 (12.6)	< 0.001
30 ~	349 (22.4)	126 (20.3)		530 (23.0)	68 (20.4)	
40 ~	382 (24.5)	179 (28.9)		630 (27.3)	89 (26.6)	
50 ~	315 (20.2)	154 (24.8)		491 (21.3)	96 (28.7)	
60 ~	218 (14.0)	81 (13.1)		196 (8.4)	39 (11.7)	
**Marital status**						
Married	1030 (66.2)	408 (65.9)	0.734	1513 (65.8)	206 (62.2)	0.397
Bereavement/divorce	71 (4.6)	33 (5.3)		193 (8.4)	32 (9.7)	
Unmarried	444 (28.6)	172 (27.8)		579 (25.2)	89 (26.9)	
Others	10 (0.6)	6 (1.0)		14 (0.6)	4 (1.2)	
**Number of cohabiting family members**						
1	204 (13.2)	81 (13.1)	0.969	234 (10.1)	41 (12.3)	0.397
2	349 (22.4)	142 (22.9)		539 (23.4)	81 (24.3)	
≥3	1002 (64.4)	396 (64.0)		1533 (66.5)	211 (63.4)	
**Employment**						
Employed	1398 (90.1)	562 (90.6)	0.687	1757 (76.2)	237 (71.0)	0.038
Unemployed	154 (9.9)	58 (9.4)		549 (23.8)	97 (29.0)	
**Total income of the entire house**						
High	769 (51.1)	294 (49.8)	0.617	1005 (46.8)	116 (38.2)	0.001
Medium	598 (39.7)	247 (41.9)		934 (43.5)	140 (46.1)	
Low	138 (9.2)	49 (8.3)		209 (9.7)	48 (15.7)	
**Residence**						
Owned house	1100 (70.9)	437 (70.8)	0.759	1630 (71.0)	236 (71.5)	0.791
Rental house	384 (24.8)	149 (24.2)		583 (25.4)	80 (24.3)	
Others	67 (4.3)	31 (5.0)		83 (3.6)	14 (4.2)	
**Welfare recipient**						
No	1525 (98.0)	606 (97.7)	0.694	2280 (98.7)	313 (93.7)	< 0.001
Yes	31 (2.0)	14 (2.3)		29 (1.3)	21 (6.3)	
**Economic conditions of current life**						
Wealthy	223 (14.3)	84 (13.6)	0.424	363 (15.8)	39 (11.7)	< 0.001
Ordinary	810 (52.3)	309 (50.1)		1226 (53.5)	142 (42.8)	
Difficult	517 (33.4)	224 (36.3)		704 (30.7)	151 (45.5)	
**Educational level**						
High	886 (57.5)	329 (53.1)	0.085	910 (39.5)	82 (24.7)	< 0.001
Low	655 (42.5)	291 (46.9)		1393 (60.5)	250 (75.3)	
**Extracurricular activities in junior or senior high school**						
None	114 (7.4)	52 (8.4)	0.094	133 (5.8)	25 (7.6)	0.031
Cultural clubs only	104 (6.8)	59 (9.6)		592 (25.8)	103 (31.6)	
Athletic clubs only	1093 (70.9)	423 (68.7)		1046 (45.6)	124 (38.0)	
Both cultural and athletic clubs	230 (14.9)	82 (13.3)		524 (22.8)	75 (22.8)	
**Living conditions at 15 years of age compared with the general public**						
Upper	35 (2.3)	12 (1.9)	0.222	61 (2.7)	10 (3.1)	< 0.001
Middle	1447 (93.6)	570 (92.3)		2137 (92.8)	285 (87.2)	
Low	64 (4.1)	36 (5.8)		104 (4.5)	32 (9.7)	
**Childhood adversity**						
Not experienced	1085 (69.6)	406 (65.5)	0.06	1530 (66.3)	187 (56.0)	< 0.001
Experienced	473 (30.4)	214 (34.5)		779 (33.7)	147 (44.0)	

Data are presented as a number (%).

*P* values were calculated using the chi-squared test for categorical variables.

### Personal or social background factors associated with obesity

To investigate further the relation between personal or social background and obesity, we conducted binomial regression analysis. Male sex was associated with obesity (OR of 2.71, with a 95% CI of 2.34–3.14, p < 0.01). Among men, no background factor was significantly associated with obesity after adjustment for age (Table 3). Among women, we adjusted for three background factors—age, welfare enrollment, and educational level—that showed a significant contribution to obesity in the stepwise procedure. In contrast to men, various personal or social background factors were significantly associated with obesity in women. Unmarried status was thus associated with obesity compared with married status (OR of 1.65, with a 95% CI of 1.21–2.25). A low household income was also associated with obesity in women compared with a high income (OR of 1.53, with a 95% CI of 1.03–2.28), as was being a welfare recipient (OR of 4.62, with a 95% CI of 2.58–8.27). In addition, difficult economic condition of current life compared with wealthy status (OR of 1.67, with a 95% CI of 1.14–2.45), a low educational level compared with a high one (OR of 1.69, with a 95% CI of 1.29–2.22), and childhood adversity (OR of 1.35, with a 95% CI of 1.07–1.72) were all associated with obesity in women.

**Table 3:** Personal or social background factors associated with obesity.

Variables	Men				Women			
	Crude OR	95% CI	Adjusted OR	95% CI	Crude OR	95% CI	Adjusted OR	95% CI
**Age (years)**								
20 ~	1		1.00		1		1.00	
30 ~	1.33	(0.96−1.83)	1.36	(0.85–2.18)	1.41	(0.94−2.12)	0.95	(0.52–1.74)
40 ~	1.72	(1.27−2.34)**	1.81	(0.85–3.84)	1.55	(1.06−2.29)*	0.67	(0.26–1.71)
50 ~	1.8	(1.31−2.46)**	1.93	(0.66–5.68)	2.15	(1.47−3.16)**	0.60	(0.16–2.30)
60 ~	1.37	(0.96−1.95)	1.49	(0.40–5.63)	2.19	(1.37−3.49)**	0.48	(0.09–2.52)
**Marital status**								
Married	1		1		1		1	
Bereavement/divorce	1.17	(0.76−1.80)	1.12	(0.73−1.72)	1.22	(0.82−1.82)	0.8	(0.52−1.24)
Unmarried	0.98	(0.79−1.21)	1.16	(0.91−1.47)	1.13	(0.87−1.47)	1.65	(1.21−2.25)**
Others	1.52	(0.55−4.20)	1.62	(0.58−4.50)	2.1	(0.68−6.44)	1.68	(0.52−5.48)
**Number of cohabiting family members**								
1	1		1		1		1	
2	0.86	(0.57−1.29)	0.86	(0.56−1.31)	0.86	(0.57−1.29)	0.85	(0.55−1.30)
≥3	0.79	(0.55−1.13)	0.89	(0.61−1.31)	0.79	(0.55−1.13)	0.89	(0.61−1.32)
**Employment**								
Employed	1		1		1		1	
Unemployed	0.94	(0.68−1.29)	0.98	(0.71−1.35)	1.31	(1.02−1.69)*	1.17	(0.90−1.52)
**Total income of the entire house**								
High	1		1		1		1	
Medium	1.08	(0.88−1.32)	1.11	(0.91−1.36)	1.3	(1.00−1.69)*	1.24	(0.96−1.62)
Low	0.93	(0.65−1.32)	0.94	(0.66−1.33)	1.99	(1.38−2.88)**	1.53	(1.03−2.28)*
**Residence**								
Owned house	1		1		1		1	
Rental house	0.98	(0.78−1.22)	1.05	(0.84−1.31)	0.95	(0.72−1.24)	0.94	(0.70−1.26)
Others	1.17	(0.75−1.81)	1.26	(0.81−1.97)	1.17	(0.65−2.09)	1.22	(0.67−2.25)
**Welfare recipient**								
No	1		1		1		1	
Yes	1.14	(0.60−2.15)	1.12	(0.59−2.11)	5.28	(2.97−9.36)**	4.62	(2.58−8.27)**
**Economic conditions of current life**								
Wealthy	1		1		1		1	
Ordinary	1.01	(0.76−1.34)	1.02	(0.77−1.36)	1.08	(0.74−1.57)	1.05	(0.72−1.53)
Difficult	1.15	(0.86−1.55)	1.15	(0.85−1.54)	2	(1.37−2.90)**	1.67	(1.14−2.45)*
**Educational level**								
High	1		1		1		1	
Low	1.18	(0.98−1.42)	1.16	(0.96−1.40)	1.99	(1.53−2.59)**	1.69	(1.29−2.22)**
**Extracurricular activities in junior or senior high school**								
None	1		1		1		1	
Cultural clubs only	1.24	(0.79−1.97)	1.24	(0.79−1.97)	0.93	(0.58−1.49)	1.19	(0.73−1.96)
Athletic clubs only	0.85	(0.60−1.20)	0.89	(0.62−1.25)	0.63	(0.40−1.01)	0.78	(0.48−1.28)
Both cultural and athletic clubs	0.78	(0.52−1.18)	0.8	(0.53−1.20)	0.76	(0.47−1.24)	0.96	(0.58−1.60)
**Living conditions at 15 years of age compared with the general public**								
Upper	1		1		1		1	
Middle	1.15	(0.59−2.23)	1.13	(0.58−2.19)	0.81	(0.41−1.61)	0.71	(0.36−1.41)
Low	1.64	(0.76−3.55)	1.57	(0.72−3.40)	1.88	(0.86−4.08)	1.25	(0.56−2.79)
**Childhood adversity**								
Not experienced	1		1		1		1	
Experienced	1.21	(0.99−1.47)	1.19	(0.98−1.45)	1.54	(1.22−1.95)**	1.35	(1.07−1.72)*

CI, confidence interval; OR, odds ratio.

The results of a binomial logistic regression analysis are shown.

Adjusted OR: Odds ratio adjusted for age in men, and for age, welfare enrollment and educational level in women.

*P < 0.05

**P < 0.01.

### Specific factors regarding childhood adversity associated with adulthood obesity in women

To examine the relation of childhood adversity to adulthood obesity in women in more detail, we divided such adversity into three categories: events experienced by parents (death, divorce, mental illness, or violence of father toward mother), maltreatment (injury due to a violent blow from a parent, inadequate food or clothing in daily life, upset caused by insults or comments of a parent), and economic difficulties. We found that, of these three categories, only maltreatment was significantly associated with adulthood obesity in women (OR of 1.59, with a 95% CI of 1.15–2.20) after adjustment for background factors—including age, welfare enrollment, educational level, and maltreatment—that showed a significant contribution to obesity in a stepwise procedure (Table 4).

**Table 4:** Factors regarding childhood adversity associated with adulthood obesity in women.

Variables	Crude OR	95% CI	Adjusted OR	95% CI
**Events experienced by parents** (death, divorce, mental illness, or violence of father toward mother)				
Not Experienced	1		1	
Experienced	1.38	(1.06−1.81)*	1.24	(0.94−1.64)
**Maltreatment** (injury due to a violent blow from a parent, inadequate food or clothing in daily life, upset caused by insults or comments of a parent)				
Not Experienced	1		1	
Experienced	1.67	(1.22−2.29)**	1.59	(1.15−2.20)**
**Economic difficulties**				
Not Experienced	1		1	
Experienced	1.48	(1.10−1.99)**	1.24	(0.91−1.68)

CI, confidence interval; OR, odds ratio.

The results of a binomial logistic regression analysis are shown.

Adjusted OR: Odds ratio adjusted for age, welfare enrollment, educational level and maltreatment.

*P < 0.05

**P < 0.01.

## Discussion

Our analysis of a large-scale community-based questionnaire survey has revealed a sex difference in the relation between obesity and personal or social background among the residents of Kobe, a representative urban area in Japan. Whereas the prevalence of obesity was higher in men than in women, personal or social background factors including marital status, economic status, educational level, and childhood adversity were significantly associated with obesity only in women. As far as we are aware, this is the first community-based study to reveal such sex-specific associations between obesity and social or personal background in Japan.

Weight stigma and weight-based disparities are often observed in employment, workplace, healthcare, education, and interpersonal settings [17]. Since women with obesity, in particular, experienced notable increases in weight discrimination even at a lower BMI than men [17], women feel more negatively about obesity than do men [17, 18], which may account for our finding that the prevalence of obesity is lower among women than among men in this study. On the other hand, the prevalence of obesity is lower among men than among women in quite a few countries [5]. The reason for gender difference in the prevalence of obesity among countries is unknown. Ethnicity, culture, and environment may be involved in its variability.

Consistent with our present findings, previous studies have uncovered a female-specific association of a lower socioeconomic status with a higher BMI in developed countries [10, 19, 20]. In Japan, two studies using the data from national surveys also showed a similar female-specific relation between obesity and low socioeconomic status including low household expenditure/income and low educational level [11, 12], which is consistent with our findings. This suggests that a female-specific relation of obesity to lower socioeconomic status observed in national surveys also appears to be applicable to urban areas in Japan. With respect to men, one of the two studies above showed no association between socioeconomic status and obesity which was defined as a BMI ≥ 25 [11]. This is consistent with our study in which obesity was also defined as a BMI ≥ 25. The other study, however, demonstrated that low educational level was inversely associated with obesity defined as a BMI ≥ 30 in men [12]. This discrepancy may be caused by the difference in the definition of obesity. A previous systematic review shows that the relationships between low socioeconomic status and obesity in women were not clear in men [10].

In the present study, unmarried status was associated with female obesity. In the two previous studies using the data of national surveys in Japan, one showed the association between marital status and female obesity [11], whereas the other showed no association between them [12]. Such discrepancy may be attributed to the differences of study conditions including ages of target subjects and the definition of obesity [11, 12]. Given that the relation of marital status to obesity varied according to sex and ethnicity in previous studies [11, 21, 22], further investigations are necessary to assess the effect of marital status to obesity.

People of higher socioeconomic status also appear to perceive overweight as a more serious condition and show a stronger motivation to lose excess body weight [23]. In particular, women often feel external pressure from the media portraying strong social norms relating to thinness and beauty [24]. Therefore, women in high socioeconomic status who can access to well-balanced diet with less energy, well-equipped exercise facilities and health care service can lose more weight than ones in low socioeconomic status. Such a female-specific settings may be responsible for the association between low socioeconomic status and obesity only in women. However, why such an association was not apparent in men has not been fully clarified. Occupational physical activity is supposed to be relatively higher in men in low socioeconomic status, which may also be one of the reasons for the prevention of obesity in men in low socioeconomic status. Other unidentified factors might limit the development of obesity in men of low socioeconomic status, the identification of which may provide a basis for preventing obesity in both men and women.

We found that childhood adversity—in particular, parental maltreatment—was associated with obesity in women. This result is consistent with previous findings that stressful psychosocial experience in childhood—especially, maltreatment or physical abuse by parents or guardians—is associated with obesity in adults [25–28]. A stronger relation between stressful psychosocial experience and obesity in women than in men has also been previously described [29]. There have been no studies demonstrating the association between childhood abuse and adulthood obesity in Japan, however. With respect to the causes of maltreatment-induce obesity, childhood abuse is reported to be associated with an increased risk of food addiction [30] or with greater use of food in response to stress in adulthood in women [31]. Together, these observations suggest that maltreatment by parents or guardians has a substantial impact on the development of obesity.

Our present study has several limitations. First, given the cross-sectional nature of the study, it is not possible to determine causality or the underlying mechanism for the observed relations between personal or social background and obesity. Second, questionnaire-based surveys can be influenced by subjective factors of the respondents. In this regard, a previous study showed a significant difference in body weight and height between self-reported and measured values [32]. And third, our study was performed among the residents of only one city in Japan. It thus remains to be established whether the current results are applicable to individuals in other regions of Japan or in other countries. Finally, it was difficult to examine the appropriateness of the questionnaire and the validity of the sample size in the present study, since this is a retrospective study using the results of a questionnaire.

## Conclusions

Analysis of a large-scale community-based questionnaire survey revealed that obesity in women is more strongly associated with personal or social background than is that in men, although the prevalence of obesity is lower in women than in men. Our study suggests that the pathogenesis of obesity is related not only to personal factors but also to social problems. Interventions to improve socioeconomic status and to prevent maltreatment in childhood may thus help to prevent obesity in women.

## Supporting information

S1 Appendix Questions concerning personal and social background in a questionnaire administered to residents of Kobe in 2018. (DOCX)

S2 Appendix An original Japanese version of a questionnaire administered to residents of Kobe in 2018. (DOCX)
