# Supplementary material for: Sex difference in the association of obesity with personal or social background among urban residents in Japan
Source: PLoS One. 2020 Nov 25;15(11):e0242105. doi: 10.1371/journal.pone.0242105 (PMC7688126; doi:10.1371/journal.pone.0242105)
Supplement: S1 Appendix — (DOCX) [file pone.0242105.s001.docx]

**S1 Appendix. Questions concerning personal and social background in a questionnaire administered to residents of Kobe in Japan (translated from the original Japanese).**

Q1 Sexuality (male, female)

Q2 Age

Q3 Marital status (married, widow/widower, divorced, unmarried)

Q4 Number of cohabiting family members (1, 2, 3...)

Q5 Health insurance (National Health Insurance, National Health Insurance Association-managed health insurance (Kyokai Kenpo), Union‐managed health insurance, Mariners’ insurance, Mutual Aid Association-managed insurance, other, none)

Q6 Employment (employed as full-time or permanent member of staff, employed as part-time or nonpermanent member of staff, self-employed, side job at home, other employed, unemployed)

Q7 Total income of the entire household before tax in 2017

Q8 Type of residence (detached house [single dwelling], detached house [multiple dwelling], public rental housing, private rental housing [single dwelling], private rental housing [multiple dwelling], rented room)

Q9 Welfare recipient (yes, no)

Q10 Economic conditions of current life (very difficult, slightly difficult, average, slightly wealthy, very wealthy)

Q11 Educational background (junior high school graduate, high school dropout, high school graduate, special training or professional school graduate, junior or technical college graduate, university dropout, university graduate, graduate school graduate, other, don’t know)

Q12 Extracurricular activities in junior or senior high school including lessons outside of the school curriculum (cultural clubs only, athletic clubs only, both cultural and athletic clubs, none, don’t know)

Q13 Living conditions at 15 years of age compared with the general public (upper, upper middle, middle, lower middle, lower)

Q14 Select all that you experienced in childhood (death of a parent, divorce of parents, mental illness of a parent, father violent toward mother, injury as the result of a violent blow from a parent, inadequate care in daily life including insufficient food or clothing, upset by insults or comments of a parent, economic difficult, none of the above)
