# Supplementary material for: Sex difference in the association of obesity with personal or social background among urban residents in Japan
Source: PLoS One. 2020 Nov 25;15(11):e0242105. doi: 10.1371/journal.pone.0242105 (PMC7688126; doi:10.1371/journal.pone.0242105)
Supplement: S2 Appendix — (DOCX) [file pone.0242105.s002.docx]

**S2 Appendix. An original Japanese version of a questionnaire administered to residents of Kobe in Japan.**

市民の健康とくらしの調査

１　あなたの性別はどちらですか。

１．男性　　　　　　　２．女性

問２　あなたは、平成30年8月１日現在、何歳ですか。

　　　満　　　　　　　 歳 （平成30年8月1日現在）

問３　あなたの婚姻状態は、次のうちのどれにあてはまりますか

１．配偶者がいる（内縁を含む）　２．死　別　 ３．離　別　 ４．未　婚　 ５．その他

問４　ご自身を含めて何人で暮らしていますか。

　　　　　　 人

問４-２【２人以上で暮らしている方】にうかがいます。同居されている方はどなたですか。

※あなたからみた続柄でお答えください。（あてはまるものすべてに○）

１．夫　　２．妻　　３．父　　４．母　　５.　配偶者の父　６．配偶者の母

７．子ども（子どもの配偶者を含む）　　　８．孫（孫の配偶者を含む）　９．祖父・祖母

10．兄弟・姉妹　　　11．その他（具体的に　　　　　　　　　　　　　　　　　　　　）

問５　あなたが現在加入している健康保険は、次のどれにあてはまりますか。（１つだけ○）

１．国民健康保険

２．全国健康保険協会管掌健康保険（協会けんぽ）　　３．組合管掌健康保険（健康保険組合）

４．船員保険　　　　　　　　　　　　　　　　　　　５．共済組合

６．その他（具体的に　　　　　　　　　　　　　　　　　　）

７．いずれも加入していない

問６　現在の就業状況について、あてはまる番号を１つ選んでください。（１つだけ○）

　※現在、育児休業などで休業中の方は、復職する時の仕事の番号を選んでください。

１．勤め（常勤・正規職員）　２．勤め（パート・アルバイト・非正規職員）

３．自営業・家業　　　　　　４．内職　　　　　５．その他の就業形態

６．仕事をしていない

【６．仕事をしていない】を選んだ方にうかがいます。

１．仕事を探している　　２．仕事を探していない（家事専業）

３．仕事を探していない（その他学生など）

次ページの問６-２へ 次ページの問７へ

問６で【１～５】を選んだ方にうかがいます。

問６－２　休日勤務は、平均して月に何日間位ありますか。（１つだけ○）

１．０日　　２．１～４日　　３．５～７日　　４．８日以上

問６で【１～５】を選んだ方にうかがいます。

問６－３　深夜労働（２２時以降）は、平均して月に何日間位ありますか。（１つだけ○）

１．０日 ２．１～４日 ３．５～９日　　　４．10～14日

５．15～19日 ６．20～24日 ７．25～31日

問６で【１～５】を選んだ方にうかがいます。

問６－４　過去１年間で平均的に残業を月に何時間程度していますか。（１つだけ○）

１．わからない ２．0～20時間 ３．21～40時間　　４．41～60時間

５．61～80時間 ６．81～100時間 ７．101時間以上

問７　世帯全体の合計収入額（年金を含みます）は、平成29年の１年間で、次のうちどれにあてはまりますか
（税引き前で）。あてはまる番号1つに○をつけてください。

１．50万円未満　　　　　　　２．50～100万円未満　　　　　 ３．100～150万円未満

４．150～200万円未満　　　　５．200～250万円未満　　　　 ６．250～300万円未満

７．300～400万円未満　　　　８．400～500万円未満　　　　 ９．500～600万円未満

10．600～700万円未満　　　　11．700～800万円未満　　　　 12．800～900万円未満

13．900～1,000万円未満　　　14．1,000万円～1,200万円未満　 15．1,200万円以上

問８　あなたが住んでいる住宅の種類はどれですか。

　　　※「借間」は、民間の賃貸住宅ではなく親類の家・部屋を借りているなど（有償・無償問わず）です。

１．持家（一戸建て） ２．持家（集合住宅）　　　　　　３．公営賃貸住宅

４．民間賃貸住宅（一戸建て） ５．民間賃貸住宅（集合住宅）　　６．借間 　７．その他

問９　あなたは現在、生活保護を受給していますか。

１．受給していない　　 ２．受給している ３．現在申請中

問10　現在の暮らしの状況を経済的にみてどう感じていますか。

１．大変苦しい　２．やや苦しい　 ３．ふつう　 ４．ややゆとりがある ５．大変ゆとりがある

問11　最後に通った（または在学中の）学校についてあてはまる番号を１つ選んでください。

　　　例　高校を卒業した場合は【３．高校卒】、高校を退学した場合は【２．高校中退】を選んでください。

１．中学校卒　　２．高校中退　　３．高校卒　　４．専修・専門学校卒　　５．短大・高専卒

６．大学中退　　７．大学卒　　８．大学院卒　　９．その他（　　　　　　　　）　10．わからない

問12　中学生か高校生の時に、部活動やクラブにはいっていましたか。（習い事を含みます。）

１．文化系のみにはいっていた　２．運動系のみにはいっていた　３．文化系・運動系ともにはいっていた

４．はいっていない　　５．覚えていない

問13　あなたが15歳当時の生活程度は、世間一般からみて、次のどれに入ると思いますか。

１．上　　　　２．中の上　　　３．中の中　　　４．中の下　　　５．下

問14　あなたが子どもの頃、経験したことがあるものすべてに○をつけてください。

１．親が亡くなった　　　　　　　　　２．親が離婚した　　　　　３．親が精神病を患っていた

４．父親が母親に暴力を振るっていた　５．親にひどく殴られてケガをした

６．食事や着替えなど、必要な世話をしてもらえなかった

７．親から傷つくことを言われたり侮辱されたりした　　　　　　　８．経済的に苦しかった

９．この中に経験したものはない
